# Mitochondrial Reprogramming Along the Gut–Brain Axis in Chemotherapy‐Induced Cognitive Impairment: A Narrative Review

**DOI:** 10.1155/omcl/5350976

**Published:** 2026-10-28

**Authors:** Amisha Malhotra, Gayathri M. Rao, Aradhana Marathe, Sharada Rai, V. Samyuktha Rani, Ajeetkumar Patil, Dinesh Upadhya, M. Chakrapani

**Affiliations:** ^1^ Department of Biochemistry Kasturba Medical College Mangalore Manipal Academy of Higher Education Manipal India manipal.edu; ^2^ Department of Pathology Kasturba Medical College Mangalore Manipal Academy of Higher Education Manipal India manipal.edu; ^3^ Manipal Institute of Applied Physics Manipal Academy of Higher Education Manipal India manipal.edu; ^4^ Department of Anatomy Kasturba Medical College Manipal Academy of Higher Education Manipal India manipal.edu; ^5^ Department of Medicine Kasturba Medical College Mangalore Manipal Academy of Higher Education Manipal India manipal.edu

## Abstract

Chemotherapy‐induced cognitive impairment (CICI), often referred to as “chemobrain,” is a frequently persistent complication that significantly affects memory, attention, executive function, and overall quality of life among cancer survivors. Although mitochondrial dysfunction, oxidative stress, neuroinflammation, and gut microbiota dysbiosis have each been implicated in its pathogenesis, the way these processes interact to drive cognitive decline remains incompletely understood. In this narrative review, we explore the current evidence supporting mitochondrial reprogramming as a central mechanistic link connecting chemotherapy‐induced alterations in the gut–brain axis to neuronal dysfunction. Here, mitochondrial reprogramming encompasses not only overt mitochondrial damage but also adaptive and maladaptive alterations in mitochondrial bioenergetics, biogenesis, mitophagy, and metabolic signaling in response to chemotherapeutic stress. We discuss how oxidative stress, impaired mitochondrial quality control, and key inflammatory and stress‐response pathways, including Nrf2/Keap1, NF‐κB, the AMPK–SIRT1–PGC‐1α axis, and mtDNA‐mediated cGAS–STING signaling, may converge to promote neuronal injury and cognitive impairment. We further examine the contribution of chemotherapy‐induced gut dysbiosis and microbiota‐derived metabolites, such as short‐chain fatty acids (SCFAs) and lipopolysaccharide (LPS), in modulating mitochondrial function, neuroinflammation, and blood–brain barrier (BBB) integrity via the gut–brain axis. Importantly, we differentiate findings derived directly from CICI models from those extrapolated from related fields such as neurodegenerative disorders, aging, and microbiome research, while highlighting current knowledge gaps and the scarcity of clinical data. Finally, we discuss promising therapeutic strategies aimed at restoring mitochondrial homeostasis and modulating the gut microbiota and outline priority areas for future research to facilitate the translation of these mechanistic insights into effective clinical interventions for cancer survivors.

**Summary**

• Mitochondrial dysfunction and gut microbiota dysbiosis have emerged as key mechanistic contributors to chemotherapy‐induced cognitive impairment (CICI).
• The gut–brain axis likely serves as a critical bidirectional link through which chemotherapy‐induced microbial dysbiosis may drive neuronal mitochondrial dysfunction.
• Several interconnected signaling pathways, including Nrf2/Keap1, NF‐κB, AMPK–SIRT1–PGC‐1α, and cGAS–STING, appear to coordinate oxidative stress, neuroinflammation, and maladaptive mitochondrial reprogramming.
• Although current evidence is primarily derived from preclinical studies, it highlights the pressing need for well‐designed longitudinal clinical investigations in cancer patients.
• Targeting mitochondrial homeostasis and modulating the gut microbiota represent promising therapeutic avenues for the prevention and mitigation of CICI.

## 1. Introduction

Chemotherapy‐induced cognitive impairment (CICI), commonly referred to as *chemobrain*, is increasingly recognized as a significant clinical complication among cancer survivors, particularly as advances in cancer treatment continue to improve long‐term survival. CICI is characterized by impairments in memory, attention, executive function, and processing speed, which may develop during chemotherapy and persist for months or even years after treatment, substantially affecting daily functioning and quality of life [1, 2]. Although the reported prevalence varies across studies, up to 75% of patients may experience cognitive impairment during treatment, and cognitive deficits can persist long after therapy completion [3]. Traditionally, the pathogenesis of CICI has been attributed to the direct neurotoxic effects of chemotherapeutic agents, including oxidative stress, neuroinflammation, impaired neurogenesis, and neuronal injury. However, these mechanisms alone do not fully explain the considerable variability in the onset, severity, and persistence of cognitive dysfunction observed among patients. Increasing evidence suggests that CICI is a multifactorial disorder involving systemic immune activation, cytokine dysregulation, vascular dysfunction, and structural as well as functional alterations within the brain [4]. Furthermore, longitudinal studies indicate that cognitive decline may begin before chemotherapy is initiated and continue to progress throughout treatment [1]. Collectively, these findings suggest that additional interconnected biological processes contribute to the development and progression of CICI.

Among these processes, mitochondrial dysfunction has emerged as a central feature of CICI pathophysiology. Neurons rely heavily on mitochondria to maintain ATP production, redox homeostasis, calcium buffering, and apoptotic signaling, making them particularly vulnerable to mitochondrial injury. Chemotherapeutic agents disrupt oxidative phosphorylation, increase reactive oxygen species (ROS) generation, damage mitochondrial DNA (mtDNA), and impair cellular bioenergetics, ultimately compromising synaptic function and neuronal survival [5]. These findings identify mitochondria as a central regulator of neuronal resilience during chemotherapy. Beyond their well‐established role in energy production, mitochondria integrate cellular metabolism, redox homeostasis, calcium signaling, and inflammatory responses, placing them at the intersection of oxidative stress, neuroinflammation, and neuronal survival.

Importantly, mitochondrial injury should not be viewed solely as a process of irreversible dysfunction. In response to chemotherapeutic stress, neurons activate adaptive mechanisms that preserve cellular bioenergetics and maintain mitochondrial homeostasis.

In this review, mitochondrial reprogramming refers to these coordinated metabolic and signaling adaptations, including alterations in mitochondrial biogenesis, mitophagy, oxidative phosphorylation, substrate utilization, and energy‐sensing pathways such as the AMP‐activated protein kinase (AMPK), sirtuin 1 (SIRT1), and peroxisome proliferator‐activated receptor gamma coactivator‐1 alpha (PGC‐1α) axis [6–10]. This concept is distinct from mitochondrial damage, which denotes structural injury to mitochondrial components, and mitochondrial dysfunction, which describes the resulting impairment in the mitochondrial function. Initially, these adaptive responses promote cellular survival by maintaining mitochondrial quality and energy homeostasis. However, persistent or severe chemotherapeutic stress may overwhelm these compensatory mechanisms, resulting in maladaptive mitochondrial remodeling, characterized by defective mitochondrial quality control, sustained oxidative stress, chronic neuroinflammation, and progressive neuronal dysfunction [6, 9, 10]. Recognizing these distinctions provides a broader framework for understanding how mitochondrial adaptations influence disease progression in CICI.

In parallel, increasing attention has focused on the gut–brain axis as an important regulator of neurological health. This bidirectional communication network integrates neural, endocrine, immune, and metabolic signaling between the gastrointestinal tract and the central nervous system. Within this axis, the gut microbiota produces a diverse range of bioactive metabolites capable of modulating neuronal signaling, immune responses, and mitochondrial function. Chemotherapy‐induced dysbiosis has been associated with alterations in microbial composition, increased intestinal permeability, systemic inflammation, and changes in microbial metabolite production. Collectively, these alterations are proposed to influence brain function through the gut–brain axis, although much of the mechanistic evidence currently derives from preclinical studies and indirect evidence from neurodegenerative disorders and broader microbiome research [11–15].

Emerging evidence further suggests that microbial metabolites, including short‐chain fatty acids (SCFAs), bile acids, and tryptophan‐derived metabolites, can influence mitochondrial metabolism, oxidative stress, and inflammatory signaling, whereas mitochondrial dysfunction may, in turn, alter immune and metabolic homeostasis. The emerging interplay between gut microbiota and mitochondrial function therefore represents a promising mechanistic framework for understanding how peripheral chemotherapy‐induced alterations may contribute to central nervous system dysfunction.

Despite these advances, direct mechanistic evidence linking chemotherapy‐induced gut dysbiosis, mitochondrial reprogramming, and cognitive impairment remains relatively limited. Much of the current understanding has been extrapolated from studies of neurodegenerative disorders, aging, cancer biology, and general microbiome research. Although these findings provide valuable biological insights, their applicability to CICI requires further validation through well‐designed experimental and clinical studies. Elucidating the temporal and mechanistic relationships between gut microbiota alterations, mitochondrial remodeling, and cognitive decline remains an important priority for future research [4, 11].

This narrative review critically examines mitochondrial reprogramming as a potential mechanistic framework linking chemotherapy‐induced mitochondrial dysfunction with gut microbiota alterations and cognitive impairment. By integrating evidence from mitochondrial biology, oxidative stress, neuroinflammation, and gut–brain axis research, we distinguish findings derived directly from CICI from those extrapolated from related neurological disorders, identify current knowledge gaps, and discuss emerging therapeutic strategies targeting mitochondrial quality control and microbiota–mitochondria interactions. A better understanding of these interconnected mechanisms may facilitate the development of more effective strategies to preserve cognitive function and improve the quality of life of cancer survivors.

## 2. Methodology

This narrative review was developed through a literature search of the PubMed, Scopus, and Web of Science databases. The literature search was conducted between February and June 2026 and included articles published from January 2010 to June 2026. Search terms were selected to capture the key concepts related to this review and included combinations of *chemotherapy-induced cognitive impairment*, *chemobrain*, *mitochondrial dysfunction*, *mitochondrial reprogramming*, *gut–brain axis*, *gut microbiota*, *oxidative stress*, *neuroinflammation*, *mitophagy*, *mitochondrial biogenesis*, and *microbiota–mitochondria crosstalk*, using Boolean operators (AND/OR) where appropriate. Original research articles, narrative reviews, systematic reviews, and metaanalyses published in peer‐reviewed journals were considered for inclusion.

Relevant studies were selected based on their scientific relevance to the objectives of this review, with particular emphasis on evidence investigating CICI, mitochondrial biology, oxidative stress, neuroinflammation, and gut microbiota alterations. Because direct evidence examining the microbiota–mitochondria axis in CICI remains limited, selected studies from related fields, including neurodegenerative diseases, aging, and cancer biology, were also included to provide a mechanistic context where it was appropriate.

The reference lists of relevant publications were manually screened to identify additional studies that contributed to the topics discussed in this review. Conference abstracts, editorials, commentaries, duplicate publications, and studies that were not directly relevant to the scope of the review were excluded. As this manuscript is a narrative review, a formal systematic review methodology, including PRISMA‐guided study selection and quantitative risk‐of‐bias assessment, was not performed. Instead, the literature was critically evaluated based on its methodological quality, scientific rigor, relevance to the review objectives, and contribution to the current understanding of mitochondrial dysfunction, gut microbiota alterations, and CICI.

## 3. Mitochondrial Homeostasis in Neuronal Function

Mitochondria are central regulators of neuronal function, integrating energy production, redox balance, calcium homeostasis, and apoptotic signaling. Neurons depend heavily on mitochondrial oxidative phosphorylation to meet the high energetic demands of synaptic transmission, membrane excitability, and axonal transport; consequently, even subtle mitochondrial impairments can significantly compromise the cognitive function [12, 13]. Within the central nervous system, mitochondria are strategically localized at synapses, where they regulate ATP generation and calcium dynamics, essential for neurotransmission and synaptic plasticity. The disruption of synaptic mitochondrial function is closely linked to impaired neuronal communication and cognitive decline [14, 15]. Mitochondrial integrity is maintained through dynamic processes of fusion and fission, which enable the adaptation to metabolic stress and facilitate the segregation of damaged components. An imbalance in these processes leads to the accumulation of dysfunctional mitochondria and increased vulnerability to neurodegenerative pathology [16, 17]. In parallel, mitochondrial quality control mechanisms, including mitophagy, biogenesis, and proteostasis preserve cellular homeostasis. Mitophagy selectively removes damaged mitochondria, whereas PGC‐1α‐mediated biogenesis replenishes mitochondrial populations in response to metabolic demand [18–20]. Mitochondria also play a critical role in buffering intracellular calcium, thereby preventing excitotoxicity and maintaining neuronal stability. Dysfunctional mitochondria fail to regulate calcium flux effectively, resulting in intracellular calcium overload and activation of neurotoxic pathways [21, 22]. Additionally, mitochondria act as both sources and targets of ROS. While physiological ROS participate in cellular signaling, excessive ROS production induces oxidative damage to lipids, proteins, and nucleic acids, a hallmark of both neurodegeneration and chemotherapy‐induced neurotoxicity [23]. Collectively, mitochondrial dysfunction represents a central pathogenic mechanism linking oxidative stress to impaired neuronal function. Unlike many other cell types, neurons possess limited regenerative capacity and depend almost exclusively on oxidative phosphorylation to meet their substantial energetic demands. Consequently, the preservation of mitochondrial homeostasis is fundamental to neuronal survival. The coordinated regulation of mitochondrial dynamics, quality‐control pathways, and bioenergetic adaptation enables neurons to respond to physiological stress. When these adaptive mechanisms are disrupted, mitochondrial dysfunction progresses to widespread impairment of the neuronal function, thereby increasing susceptibility to cognitive decline.

## 4. Neurobiology of CICI

CICI is a multifactorial neurological disorder resulting from complex structural, cellular, and molecular alterations within the central nervous system. Among brain regions, the hippocampus, which is critical for learning and memory, is particularly vulnerable to chemotherapy‐induced injury. Chemotherapeutic agents such as methotrexate and 5‐fluorouracil impair hippocampal function by disrupting neurogenesis and synaptic plasticity, ultimately leading to measurable cognitive deficits [24, 25]. Although the underlying mechanism differs among chemotherapeutic agents, these neurotoxic effects arise through a combination of direct neuronal injury and indirect systemic responses.

A hallmark feature of CICI is impaired adult neurogenesis, particularly within the dentate gyrus of the hippocampus. Chemotherapy inhibits the proliferation and differentiation of neural progenitor cells, resulting in reduced neuronal replacement and compromised cognitive flexibility and spatial memory [24–26]. Additionally, chemotherapeutic agents affect oligodendrocyte lineage cells, contributing to white matter damage and disrupted neural connectivity, key factors underlying cognitive decline in cancer survivors [26–28].

These structural and cellular alterations are closely linked to mitochondrial dysfunction, which represents another central mechanism in CICI. Chemotherapy also triggers systemic immune activation, leading to increased production of proinflammatory cytokines such as interleukin‐6 (IL‐6), tumor necrosis factor‐alpha (TNF‐α), and interleukin‐1β (IL‐1β) (Table 1). These cytokines can cross or disrupt the blood–brain barrier (BBB) or activate resident immune cells, including microglia and astrocytes, thereby promoting a sustained neuroinflammatory state.

**Table 1:** Major mechanisms underlying chemotherapy‐induced cognitive impairment and their contribution to mitochondrial reprogramming along the gut–brain axis.

Mechanism	Key features	Representative evidence	Impact on cognition	Role in mitochondrial reprogramming
Neuroinflammation	Increased IL‐6, TNF‐α, and IL‐1β; microglial activation; chronic neuroimmune signaling	Chemotherapy‐induced cytokine elevation and microglial activation [29–34]	Synaptic dysfunction, neuronal injury, and impaired memory	Proinflammatory cytokines impair mitochondrial respiration, increase mtROS, and disrupt ATP production
Oxidative stress	Excess ROS generation; lipid, protein, and mtDNA oxidation	Experimental models of chemotherapy‐induced oxidative injury [23, 31, 32, 34, 35]	Impaired neuronal signaling and memory deficits	Oxidative damage disrupts oxidative phosphorylation and mitochondrial quality control
Mitochondrial dysfunction/Reprogramming	Altered oxidative phosphorylation; ATP depletion; impaired mitochondrial biogenesis; and defective mitophagy	Mitochondrial injury following 5‐FU and cisplatin and doxorubicin exposure [36–39]	Reduced neuronal resilience and cognitive decline	Central integrative mechanism linking oxidative stress, inflammation, and metabolic adaptation
Impaired neurogenesis	Reduced hippocampal neural progenitor proliferation and decreased synaptic plasticity	Methotrexate and 5‐FU experimental studies [24–26]	Learning and memory impairment	Energy deficiency limits neuronal regeneration and synaptic remodeling
White matter injury	Oligodendrocyte damage; demyelination; and axonal degeneration	Neuroimaging and experimental studies [26, 28]	Reduced processing speed and executive dysfunction	Mitochondrial dysfunction compromises myelin maintenance and axonal energy supply
Gut microbiota dysbiosis	Loss of microbial diversity; reduced SCFA‐producing bacteria; and increased intestinal permeability	Chemotherapy‐associated dysbiosis studies [40–45]	Neuroinflammation and impaired cognition through the gut–brain axis	Reduced microbial metabolites impair mitochondrial bioenergetics and increase inflammatory signaling
Gut–brain axis dysregulation	Altered vagal signaling; HPA‐axis activation; and blood–brain barrier disruption	Preclinical gut–brain communication studies [40, 41, 45–47]	Cognitive dysfunction and altered stress responses	Connects peripheral microbial alterations with central mitochondrial dysfunction
Neurotransmitter dysregulation	Altered serotonin, dopamine, glutamate, and GABA signaling	Clinical and experimental studies [3, 6, 48]	Attention deficits, mood disturbances, and impaired cognition	Mitochondrial dysfunction disrupts neurotransmitter synthesis and synaptic transmission

Persistent microglial activation impairs synaptic function, inhibits neurogenesis, and contributes to neuronal cell death [29, 30]. Oxidative stress further exacerbates these neurobiological alterations. Chemotherapeutic agents elevate ROS levels beyond the capacity of endogenous antioxidant defenses, resulting in lipid peroxidation, protein oxidation, and DNA damage. Neurons are particularly vulnerable due to their high metabolic demand and limited regenerative capacity. The interplay between oxidative stress and inflammation creates a self‐perpetuating cycle that amplifies neuronal damage and cognitive decline [31, 32].

In addition, alterations in neurotransmitter systems contribute to the cognitive deficits observed in CICI. Dysregulation of dopaminergic, serotonergic, and glutamatergic signaling pathways affects mood, attention, and executive function. Neuroimaging studies further demonstrate reduced functional connectivity in key cognitive networks following chemotherapy [32].

Collectively, impaired neurogenesis, neuroinflammation, oxidative stress, white matter damage, and neurotransmitter dysregulation interact to produce widespread neuronal dysfunction. Understanding these mechanisms provides a foundation for investigating upstream regulators, including mitochondrial dysfunction and gut–brain axis interactions, which are explored in subsequent sections.

## 5. Cellular Senescence, Oxidative Stress, and Mitochondrial Dysfunction in Neurodegeneration

Cellular senescence represents a stress‐induced irreversible cell‐cycle arrest triggered by factors such as oxidative stress, DNA damage, mitochondrial dysfunction, and chemotherapeutic exposure [49–51]. While senescence initially functions as a protective mechanism limiting the proliferation of damaged cells, its persistent accumulation contributes to tissue dysfunction, aging, and chronic disease progression [52].

A key feature of senescent cells is the senescence‐associated secretory phenotype (SASP), characterized by the release of proinflammatory mediators, including IL‐6, IL‐1β, and TNF‐α [50, 53]. These factors profoundly remodel the tissue microenvironment and promote a state of chronic, low‐grade inflammation (“inflammaging”), thereby reinforcing senescence in neighboring cells and sustaining inflammatory signaling networks. Mitochondrial dysfunction is a central driver linking oxidative stress to the onset and persistence of cellular senescence. Elevated ROS production, mtDNA damage, and impaired oxidative phosphorylation disrupt redox homeostasis and activate senescence‐associated pathways [54].

In parallel, mitochondrial stress promotes the release of damage‐associated molecular patterns (DAMPs), which activate innate immune signaling and amplify inflammatory responses [51]. This establishes a feed‐forward cycle in which oxidative damage and mitochondrial impairment mutually reinforce each other (Figure 1).

**Figure 1:**
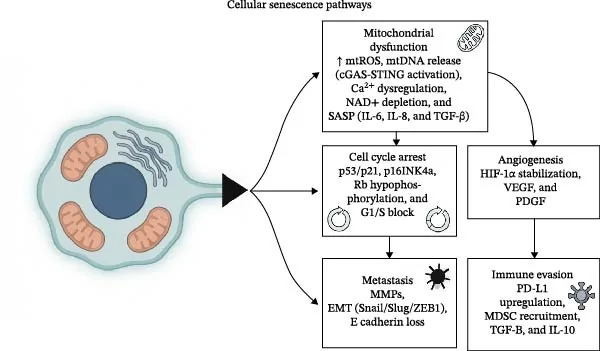
Mitochondrial senescence is associated with increased ROS production, mtDNA release, Ca^2+^ imbalance, and activation of the senescence‐associated secretory phenotype (SASP). These changes promote cell‐cycle arrest, angiogenesis, metastasis, and immune evasion through p53/p21 signaling, VEGF/HIF‐1α activation, EMT induction, and proinflammatory cytokine release. cGAS‐STING, cyclic GMP–AMP synthase‐stimulator of interferon genes; EMT, epithelial‐to‐mesenchymal transition; HIF‐1α, hypoxia‐inducible factor 1‐alpha; MDSC, myeloid‐derived suppressor cells; MMPs, matrix metalloproteinases; mtDNA, mitochondrial DNA; mtROS, mitochondrial reactive oxygen species; NAD+, nicotinamide adenine dinucleotide; PDGF, platelet‐derived growth factor; PD‐L1, programmed death‐ligand 1; SASP, senescence‐associated secretory phenotype; Slug (SNAI2), transcription factor that drives EMT and promotes metastasis by suppressing E‐cadherin and enhancing cell motility; Snail (SNAI1), transcription factor that drives EMT and promotes metastasis by suppressing E‐cadherin and enhancing cell motility; TGF‐β, transforming growth factor beta; VEGF, vascular endothelial growth factor.

In the central nervous system, the accumulation of senescent neurons and glial cells is closely associated with neuroinflammation and cognitive decline [50]. Senescent astrocytes and microglia adopt proinflammatory phenotypes that disrupt synaptic homeostasis and compromise BBB integrity [55, 56]. Furthermore, mtDNA leakage and subsequent activation of cytosolic DNA‐sensing pathways, particularly cGAS–STING, sustain neuroinflammatory signaling and contribute to disease progression [57].

Chemotherapy further exacerbates these processes by accelerating cellular senescence through oxidative stress and mitochondrial dysfunction [58]. This results in endothelial and neural injury, BBB disruption, glial activation, and persistent cognitive impairment, supporting the concept of chemotherapy‐induced cognitive dysfunction as an accelerated aging phenotype rather than a transient toxic effect [58, 59]. At the molecular level, longevity‐associated signaling pathways, including SIRT1 and AMPK, play pivotal roles in regulating the redox balance, mitochondrial function, and metabolic adaptation. Dysregulation of these pathways promotes the persistence of senescent cells and enhances susceptibility to age‐related neurodegenerative changes.

Overall, the interplay between oxidative stress, mitochondrial dysfunction, and cellular senescence establishes a self‐perpetuating cycle characterized by chronic inflammation, metabolic imbalance, and neuronal damage. This integrated network forms a key mechanistic axis underlying CICI, with redox signaling exerting a direct influence on mitochondrial biogenesis, dynamics, and metabolic adaptation.

## 6. Chemotherapy‐Induced Mitochondrial Dysfunction

Mitochondrial dysfunction is a central mechanism underlying CICI. Although chemotherapeutic agents target rapidly dividing cancer cells, they exert off‐target effects on postmitotic neurons, which rely heavily on mitochondrial integrity for energy production and synaptic function [60, 61]. A primary consequence of chemotherapy is the disruption of oxidative phosphorylation, leading to reduced ATP production. Agents such as doxorubicin impair the electron transport chain, resulting in electron leakage and excessive generation of ROS [36]. This promotes oxidative damage to mitochondrial lipids, proteins, and mtDNA, establishing a self‐perpetuating cycle of mitochondrial dysfunction [37, 62, 63]. mtDNA is particularly vulnerable due to its proximity to the electron transport chain and the lack of protective histones. Its damage impairs mitochondrial biogenesis and compromises neuronal energy metabolism, contributing to cognitive decline [64].

The mechanisms by which chemotherapy disrupts mitochondrial function are not uniform and vary according to the class of the chemotherapeutic agent. For example, anthracyclines such as doxorubicin mainly promote oxidative mitochondrial injury by increasing ROS production [36], whereas platinum compounds are more commonly associated with mtDNA damage, calcium imbalance, and mitochondrial membrane dysfunction [62]. Antimetabolites, including methotrexate and 5‐fluorouracil, predominantly interfere with mitochondrial energy metabolism and hippocampal neurogenesis [37, 60]. These mechanistic differences may help explain why the severity and clinical presentation of CICI differ among patients and treatment regimens [60, 61].

Chemotherapy also disrupts mitochondrial dynamics, shifting the fusion–fission balance toward fragmentation [38]. This leads to the accumulation of dysfunctional mitochondria, reduced bioenergetic efficiency, and activation of apoptotic pathways [65]. Impaired mitophagy further exacerbates this process by limiting the clearance of damaged mitochondria. Mitochondrial dysfunction additionally promotes apoptosis through membrane permeabilization and release of cytochrome *c*, triggering caspase activation and neuronal loss [65, 66]. Alongside these direct effects, chemotherapy‐induced inflammatory mediators suppress mitochondrial respiration and biogenesis, contributing to metabolic imbalance and energy deficits [67]. Collectively, these alterations reinforce oxidative stress, mitochondrial failure, and neuronal injury, ultimately promoting cellular senescence and neurodegeneration in CICI.

## 7. Mitochondrial Biogenesis and Metabolic Regulation in CICI

Mitochondrial biogenesis and metabolic regulation are essential adaptive processes that preserve neuronal energy homeostasis. In CICI, disruption of these processes contributes to mitochondrial dysfunction and increased neuronal vulnerability, increasing susceptibility to bioenergetic failure and cognitive dysfunction [33, 68, 69]. PGC‐1α is a master regulator of mitochondrial biogenesis, coordinating genes involved in oxidative phosphorylation and redox balance [69]. In neurons, it supports mitochondrial density and ATP production required for synaptic function, as activation of PGC‐1α represents the earliest responsiveness, which promotes mitochondrial renewal and restoration of ATP production following the cellular injury.

Dysregulation of PGC‐1α signaling is associated with impaired bioenergetics and neurodegeneration [70]. Its activity is controlled by metabolic sensors such as AMPK and SIRT1. AMPK activates PGC‐1α in response to energy depletion, while SIRT1 enhances its activity through NAD^+^‐dependent deacetylation. Together, the AMPK–SIRT1–PGC‐1α axis functions as a central metabolic sensor that coordinates mitochondrial reprogramming by integrating cellular energy status with mitochondrial biogenesis, oxidative metabolism, and stress adaptation [69, 71].

In addition, SIRT3 cooperates with antioxidant pathways such as Nrf2/Kelch‐like ECH‐associated protein 1to maintain mitochondrial redox homeostasis by enhancing the expression of enzymes involved in ROS detoxification, oxidative metabolism, and antioxidant defense within mitochondria. Reduced SIRT3 activity exacerbates oxidative stress and increases neuronal vulnerability to injury [72, 73]. Mitochondrial quality control relies on a delicate balance between biogenesis and mitophagy. Disruption of this balance leads to the accumulation of damaged mitochondria, heightened oxidative stress, and impaired energy metabolism [33, 73, 74]. Chemotherapeutic agents disrupt these regulatory mechanisms by impairing AMPK signaling, suppressing mitochondrial biogenesis, and causing ATP depletion [33, 68].

Under sustained chemotherapeutic stress, neurons undergo metabolic reprogramming characterized by a subtle shift from oxidative phosphorylation to glycolysis. Although this transition initially serves as an adaptive response to maintain ATP production, prolonged reliance on glycolysis reduces energy efficiency, exacerbates oxidative damage, and accelerates neuronal dysfunction [75].

Taken together, mitochondrial biogenesis, mitophagy, and metabolic regulation represent particularly an integrated adaptive network that determines neuronal resilience following chemotherapy [39, 71]. Persistent dysregulation promotes cellular senescence, further driving neuroinflammation and cognitive decline.

## 8. Interplay Between Oxidative Stress, Neuroinflammation, and Mitochondrial Dysfunction in CICI

Oxidative stress and neuroinflammation are central, interconnected mechanisms driving CICI. Chemotherapeutic agents disrupt cellular redox homeostasis, leading to excessive production of reactive oxygen and nitrogen species (ROS/RNS). When these exceed endogenous antioxidant capacity, oxidative damage to lipids, proteins, and nucleic acids occurs, particularly in neurons, which are highly metabolically active but have limited regenerative capacity [34, 76].

Mitochondria are both major sources and targets of this oxidative imbalance. Chemotherapy‐induced impairment of the electron transport chain promotes electron leakage and further ROS generation, creating a self‐amplifying cycle of mitochondrial damage. mtDNA, due to its proximity to ROS generation sites and the lack of protective histones, is especially vulnerable. Its damage impairs mitochondrial biogenesis and energy production, exacerbating neuronal energy deficits [35, 77] (Table 2).

**Table 2:** Mitochondrial alterations underlying chemotherapy‐induced cognitive impairment and their contribution to mitochondrial reprogramming.

Stage of mitochondrial response	Major cellular events	Representative mechanisms	Functional consequence in CICI	References
Early adaptive response	Increased mitochondrial metabolic flexibility to maintain ATP production under chemotherapy‐induced stress	Transient activation of antioxidant defenses, mitochondrial biogenesis, and metabolic adaptation	Preserves neuronal energy homeostasis and delays cellular injury	[9–11, 69–71]
Persistent oxidative stress	Excess mitochondrial ROS production exceeds antioxidant capacity	Oxidative damage to lipids, proteins and mtDNA; impaired electron transport chain activity	Reduced ATP production and initiation of neuroinflammation	[23, 31, 32, 34–36]
Loss of mitochondrial quality control	Impaired mitochondrial dynamics and defective mitophagy	Excess mitochondrial fission, reduced fusion, and accumulation of dysfunctional mitochondria	Progressive mitochondrial dysfunction and neuronal vulnerability	[11, 38, 39]
Maladaptive mitochondrial reprogramming	Failure of adaptive bioenergetic remodeling with sustained metabolic dysfunction	Persistent oxidative phosphorylation impairment, chronic ROS generation, and altered cellular metabolism	Synaptic dysfunction, impaired neuroplasticity, and cognitive decline	[39, 78–80]
Neuronal degeneration	Activation of mitochondria‐mediated apoptosis and chronic neuroinflammation	Cytochrome *c* release, caspase activation, and inflammatory signaling	Irreversible neuronal loss and persistent cognitive impairment	[26, 32, 34, 35, 66]

Under normal physiological conditions, intracellular redox balance is maintained by endogenous antioxidant defense systems, with the nuclear factor erythroid 2‐related factor 2 (Nrf2)/(Keap1) pathway serving as a key regulator of this response. Activation of Nrf2 induces the expression of antioxidant and cytoprotective genes involved in glutathione (GSH) metabolism, ROS detoxification, and the preservation of mitochondrial homeostasis [73, 74] (Figure 2).

**Figure 2:**
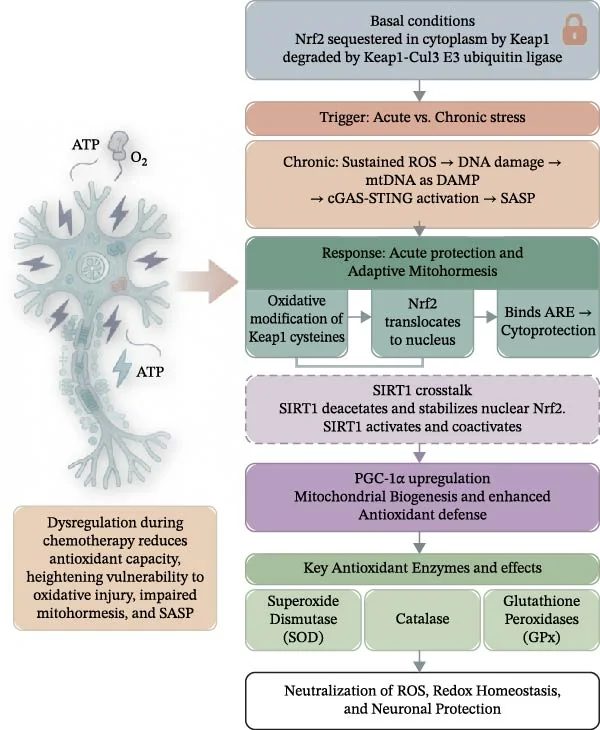
Nrf2–Keap1 antioxidant pathway and SIRT1 crosstalk in neurons. Basal conditions keep Nrf2 sequestered and degraded by Keap1. Chemotherapy‐induced mitochondrial ROS and mtDNA release as damage‐associated molecular patterns (DAMPs) activate cGAS‐STING signaling and SASP. Nrf2 translocates to the nucleus, inducing cytoprotective genes (SOD, catalase, and GPx). SIRT1 crosstalk stabilizes Nrf2 and upregulates PGC‐1α, driving mitochondrial reprogramming. Dysregulation increases oxidative injury and neuroinflammation in chemotherapy‐induced cognitive impairment.

However, excessive oxidative stress induced by chemotherapy can impair Nrf2 signaling, reducing the capacity of cells to counteract ROS accumulation. Thus, it results in an imbalance that promotes mitochondrial dysfunction, enhances inflammatory signaling, and increases neuronal susceptibility to injury, thereby contributing to the development and progression of CICI.

Neuroinflammation develops in parallel with oxidative stress and further amplifies neuronal injury. Chemotherapy‐induced systemic inflammation increases the circulating levels of proinflammatory cytokines, including IL‐6, TNF‐α, and IL‐1β [81]. These cytokines can cross or disrupt the BBB and activate microglia and astrocytes. Activated microglia adopt a proinflammatory phenotype, sustaining the production of cytokines and neurotoxic mediators, thereby perpetuating chronic neuroinflammation [82, 83]. This inflammatory response is further exacerbated by depletion of intracellular GSH, which is one of the principal endogenous antioxidants in neurons, thereby reducing GSH levels, limiting the ability of cells to neutralize ROS and reinforcing oxidative stress, mitochondrial dysfunction, and inflammatory signaling during chemotherapy.

A key mechanistic link between oxidative stress and inflammation is the activation of regulatory redox‐sensitive signaling pathways, particularly nuclear factor kappa B (NF‐κB) and Nrf2/Keap1. ROS‐mediated cytokine activation enhances production, while inflammatory mediators further impair mitochondrial function, reinforcing oxidative stress and neuronal damage in a feed‐forward loop that amplifies oxidative damage and neuronal dysfunction [66, 84].

Oxidative stress and inflammation also compromise BBB integrity by disrupting tight junction proteins, increasing permeability, and allowing peripheral immune cells and toxic molecules to enter the CNS. This further aggravates neuroinflammation and neuronal injury [84, 85].

Taken together, oxidative stress and neuroinflammation establish a self‐reinforcing network of mitochondrial dysfunction during chemotherapy. Impaired antioxidant responses, with systemic inflammation ultimately driving neuronal damage and cognitive impairment, highlight these interconnected pathways as promising therapeutic targets for mitigating CICI.

## 9. Gut–Brain Axis Signaling in CICI

The gut–brain axis is a bidirectional communication network linking the gastrointestinal tract and central nervous system through neural, endocrine, immune, and metabolic pathways [46]. This system enables the gut microbiota to influence cognitive function, behavior, and brain activity, thereby playing a critical role in neurological homeostasis as well as CICI [40, 41]. Neural communication is primarily mediated by the vagus nerve, which transmits signals derived from microbial activity and intestinal stimuli to the brain. Microbiota modulate vagal signaling either directly through metabolites or indirectly via enteroendocrine cells, influencing neurotransmission and neural function [86, 87].

The endocrine component involves the hypothalamic–pituitary–adrenal (HPA) axis, which responds to stress and inflammation by releasing glucocorticoids. Dysregulation of this axis alters gut microbial composition and increases susceptibility to neuroinflammation, linking peripheral stress responses to cognitive outcomes [47]. Immune signaling is another key component as gut microbiota regulates cytokine production and immune homeostasis. Dysbiosis promotes systemic inflammation, allowing cytokines to cross or disrupt the BBB and activate microglia, thereby contributing to neuroinflammation and cognitive impairment [88, 89].

Metabolically, the gut microbiota produces bioactive compounds, including SCFAs, tryptophan metabolites, bile acids, and neurotransmitter precursors. These metabolites regulate mitochondrial function, inflammation, and neurotransmitter synthesis. Butyrate supports neuronal health by enhancing mitochondrial bioenergetics and modulating gene expression [90, 91].

In addition to their immunomodulatory effects, these microbial metabolites play an important role in maintaining mitochondrial bioenergetics, regulating oxidative stress, and supporting mitochondrial quality control, thereby providing a functional link between gut microbial homeostasis and neuronal health in CICI [41, 90, 91]. Disruption of intestinal barrier integrity (“leaky gut”) permits microbial components to enter systemic circulation, promoting inflammation and increasing BBB permeability; this facilitates the entry of inflammatory mediators into the CNS, exacerbating neuroinflammation [92, 93].

Among these microbial products, lipopolysaccharide (LPS), a major component of the outer membrane of Gram‐negative bacteria, has been widely implicated in promoting systemic inflammation. Increased circulating LPS can activate Toll‐like receptor 4 (TLR4)‐mediated signaling, further amplifying inflammatory responses and contributing to neuroimmune activation [41, 47].

Emerging evidence suggests that the effects of chemotherapy on the gut microbiota differ depending on the chemotherapeutic agent, although a consistent microbial signature associated with CICI has not yet been identified [42, 48, 94, 95].

Experimental studies have shown that treatment with 5‐fluorouracil is often accompanied by a decline in beneficial SCFA‐producing bacteria, including *Lactobacillus* and *Bifidobacterium*, together with an increase in potentially proinflammatory taxa such as *Escherichia/Shigella* and *Enterococcus* [42, 94].

Methotrexate has similarly been associated with reduced microbial diversity, alterations in the abundance of *Bacteroides* and members of the *Lachnospiraceae* family, and disruption of intestinal barrier integrity. Platinum‐based chemotherapeutic agents have also been reported to alter gut microbial composition, particularly by reducing butyrate‐producing bacteria, although the specific taxa affected vary across studies [42, 94, 95].

Despite these differences, a common observation is the loss of beneficial microbial communities and their metabolites, particularly SCFAs, accompanied by increased intestinal permeability and systemic inflammation. These findings suggest that while the taxonomic changes induced by chemotherapy are heterogeneous, the resulting functional consequences appear to be more consistent and may contribute to the development of CICI [42, 48, 94, 95].

Overall, disruption of gut–brain axis signaling during chemotherapy contributes to cognitive impairment through integrated neural, immune, endocrine, and metabolic mechanisms. Emerging evidence suggests that CICI‐induced dysbiosis may promote neuroinflammation and impair mitochondrial function; however, the current mechanistic understanding is all based upon preclinical studies. Further, well‐designed longitudinal clinical studies are required to clarify these relationships, establish causality, and determine whether modulation of the gut microbiota could serve as a viable therapeutic strategy for patients experiencing cognitive impairment.

## 10. Gut Microbiota Alterations in Chemotherapy

Chemotherapy disrupts gut microbial homeostasis, leading to dysbiosis characterized by reduced microbial diversity and an altered community composition [94, 95]. Although specific microbial changes vary according to the chemotherapeutic regimen, treatment is generally associated with a decline in beneficial bacteria such as Lactobacillus and Bifidobacterium, together with an increase in opportunistic and proinflammatory microorganisms [43, 44].

This imbalance compromises key microbiota functions, including maintenance of intestinal barrier integrity, immune regulation, and the production of metabolites such as SCFAs. Loss of butyrate‐producing bacteria is particularly detrimental as butyrate plays a critical role in epithelial integrity, suppressing inflammatory signaling and mitochondrial function [96, 97]. Reduced production of these metabolites may also impair the host energy metabolism and mitochondrial homeostasis, providing a potential mechanistic link between chemotherapy‐induced dysbiosis and neuronal dysfunction [44, 97].

Chemotherapy‐induced epithelial damage further increases intestinal permeability by disrupting tight junction proteins, allowing bacterial components such as LPS to enter circulation. This promotes systemic inflammation and immune activation, which can propagate to the brain via the gut–brain axis [98]. Among these microbial products, LPS is considered a major component of the outer membrane of Gram‐negative bacteria and has been widely implicated in promoting systemic inflammation through TLR4‐mediated signaling, thereby amplifying neuroimmune responses [96–98].

Dysbiosis also alters microbial metabolism, leading to changes in bioactive compounds such as bile acids, neurotransmitter precursors, and microbial toxins. These alterations influence immune responses, mitochondrial homeostasis, and neurochemical signaling, thereby contributing to systemic toxicity and increasing susceptibility to cognitive impairment [99]. Moreover, the gut microbiota modulates chemotherapeutic drug metabolism and immune responses. Dysbiosis may therefore reduce treatment efficacy and exacerbate toxicity [99–101].

Collectively, chemotherapy‐induced dysbiosis contributes to systemic inflammation, metabolic disruption, and neuroinflammation, thereby playing a central role in CICI pathogenesis. Understanding how these alterations influence mitochondrial function may provide new insights into the mechanisms underlying CICI.

## 11. Microbiota–Mitochondria Crosstalk in CICI

The interaction between the gut microbiota and mitochondria represents a key interface linking metabolism, immunity, and neurobiology. This relationship is supported by their shared evolutionary origin, reflected in interconnected signaling metabolic pathways that influence both host physiology and disease progression [102, 103].

Gut microbiota regulate mitochondrial function primarily through the production of bioactive metabolites. SCFAs serve as substrates for oxidative metabolism, supporting ATP generation and regulating redox balance and mitochondrial biogenesis, including the AMPK‐SIRT1‐PGC‐1α axis [104].

Tryptophan metabolites and bile acids further influence mitochondrial metabolism and inflammatory pathways through signaling mechanisms such as aryl hydrocarbon receptor (AhR) activation and nuclear receptors, including FXR and TGR5 [105–107].

Conversely, mitochondria influence immune responses that shape the gut microbial composition. Through mitochondrial ROS (mtROS) production and inflammasome activation, mitochondria regulate inflammatory signaling and microbial homeostasis [108, 109]. Oxidative stress serves as a central link in this bidirectional interaction (Figure 3).

**Figure 3:**
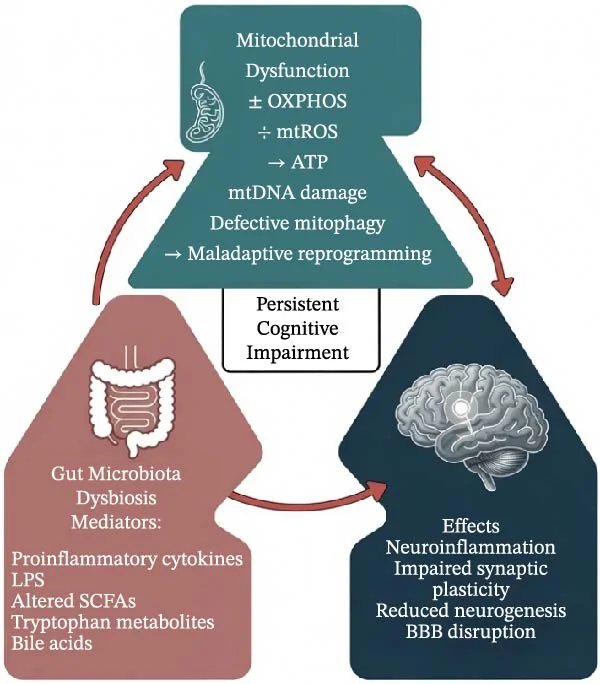
Mitochondrial–gut–brain axis in chemotherapy‐induced cognitive impairment. Mitochondrial dysfunction (impaired OXPHOS, ↑ mtROS, mtDNA damage, and defective mitophagy) interacts with gut dysbiosis (proinflammatory cytokines, LPS, and altered SCFAs) to drive neuroinflammation, BBB disruption, and persistent cognitive impairment via self‐reinforcing vicious cycles.

Dysbiosis enhances the production of microbial toxins and inflammatory mediators, which increase mitochondrial ROS generation. Excess ROS further damages mitochondrial function, reinforcing a cycle of metabolic and inflammatory dysfunction [108, 110]. Disruption of this crosstalk reduces neuronal resilience and increases susceptibility to cognitive impairment, highlighting the microbiota–mitochondria axis as a crucial mechanism in CICI [78, 79].

## 12. Mitochondrial Reprogramming: An Integrated Mechanistic Framework for CICI

Mitochondrial reprogramming refers to adaptive changes in cellular energy metabolism in response that enable cells to maintain energy homeostasis under conditions of physiological or pathological stress. In the context of CICI, these adaptive responses integrate the direct effects of chemotherapeutic agents with systemic alterations arising from gut dysbiosis, chronic inflammation, and oxidative stress, ultimately influencing survival and cognitive function [78, 79].

Current evidence suggests that chemotherapy‐induced alterations in gut microbiota reduce the production of key metabolites and promote persistent inflammatory signaling, thereby affecting mitochondrial homeostasis and cellular energy metabolism. Although these mitochondrial adaptations initially serve to preserve cellular function under stress, prolonged and repeated chemotherapeutic exposure may overwhelm these compensatory responses. Which, as a result, shifts the adaptive mitochondrial remodeling to a maladaptive state characterized by impaired oxidative phosphorylation, defective mitochondrial quality control, sustained oxidative stress, and neuronal dysfunction [80, 111–113].

This metabolic shift is further exacerbated by mtDNA damage and impaired electron transport chain function. Thus, mitochondrial reprogramming represents a convergence point where dysbiosis, metabolic disruption, and neuroinflammation interact to drive cognitive decline, and much of the current mechanistic understanding is derived from preclinical studies. Future longitudinal clinical investigations integrating microbiome profiling, mitochondrial biomarkers, and comprehensive cognitive assessment will be essential to validate these mechanisms, establish their clinical relevance, and facilitate the development of targeted therapeutic strategies for patients with CICI.

## 13. Therapeutic Implications

Recognition of mitochondrial dysfunction and gut microbiota dysbiosis as central contributors to CICI has enabled the development of mechanism‐based therapeutic strategies. While clinical management primarily focuses on supportive care and cognitive rehabilitation, increasing evidence suggests that targeting mitochondrial homeostasis and gut microbial composition may provide sustained neuroprotective benefits. However, emerging evidence suggests that restoring metabolic and microbial homeostasis may provide more effective and sustained benefits if translated into routine and clinical practice.

Targeting mitochondrial dysfunction represents a key therapeutic strategy. Antioxidants designed to reduce oxidative stress have shown promise in experimental models by attenuating chemotherapy‐induced neuronal damage [114, 115]. Compounds such as coenzyme Q10, alpha‐lipoic acid, and mitochondria‐targeted antioxidants (e.g., MitoQ) enhance mitochondrial bioenergetics, reduce ROS, and preserve neuronal function [114–116]. In addition, therapeutic approaches aimed at enhancing mitochondrial biogenesis through pathways involving AMPK‐SIRT1‐PGC‐1α may improve cellular energy balance and improve neuronal resilience, although clinical evidence remains limited [117].

Modulation of the gut microbiota offers another important therapeutic avenue. Probiotics and prebiotics have been shown to restore microbial diversity, enhance the production of beneficial metabolites such as SCFAs, and reduce systemic inflammation, thereby influencing brain function via the gut–brain axis [118]. A randomized controlled trial suggests that probiotic supplementation may improve cognitive performance in patients with breast cancer receiving chemotherapy [119].

Similarly, fecal microbiota transplantation (FMT) has emerged as a potential strategy for restoring microbial homeostasis, although its safety, efficacy, and long‐term outcomes in oncology patients require further investigations before widespread clinical application.

Dietary interventions further support both mitochondrial function and microbial balance. Diets rich in antioxidants, polyphenols, and omega‐3 fatty acids have been associated with improved mitochondrial efficiency and reduced oxidative stress and favorable alterations in microbial composition [120]. Polyphenols may modulate mitochondrial signaling pathways while promoting the growth of the essential gut microorganism, suggesting a dual mechanism through which dietary strategies benefit in mitigating chemotherapy‐related toxicity (Table 3).

**Table 3:** Therapeutic strategies targeting mitochondrial reprogramming along the gut–brain axis in chemotherapy‐induced cognitive impairment.

Mechanistic driver	Therapeutic target	Representative intervention	Potential clinical outcome	References
Excess mitochondrial oxidative stress	Restore redox homeostasis and mitochondrial bioenergetics	MitoQ, CoQ10, and α‐lipoic acid	Reduced neuronal oxidative injury and improved mitochondrial function	[114, 115]
Impaired mitochondrial biogenesis and ATP production	Enhance mitochondrial biogenesis (AMPK–SIRT1–PGC‐1α pathway)	PGC‐1α activators and exercise	Improved neuronal energy metabolism and resilience	[69, 71, 117, 121, 122]
Gut microbiota dysbiosis	Restore microbial diversity and SCFA production	Probiotics and prebiotics	Reduced neuroinflammation and improved gut–brain communication	[40, 41, 45, 94, 118, 119]
Loss of microbial metabolites (SCFAs)	Reestablish microbiota‐derived metabolic signaling	Probiotics, dietary fiber, and polyphenol‐rich diet	Improved mitochondrial metabolism and reduced inflammation	[98, 99, 104, 118]
Chronic neuroinflammation	Suppress inflammatory signaling and secondary mitochondrial injury	Antiinflammatory therapies and vagus nerve stimulation	Preservation of neuronal function and reduced cognitive decline	[29, 32, 82, 123, 124]
Impaired gut–brain communication	Restore gut–brain axis signaling	Fecal microbiota transplantation (FMT) and microbiota‐directed therapies	Improved microbial homeostasis and neuronal resilience	[40, 41, 45, 78, 80]
Multisystem metabolic dysfunction	Simultaneously target mitochondrial and microbial homeostasis	Exercise combined with dietary interventions	Long‐term preservation of cognitive function and improved quality of life	[120–122]

Abbreviations: AMPK, AMP‐activated protein kinase; ATP, adenosine triphosphate; CoQ10, coenzyme Q10; FMT, fecal microbiota transplantation; PGC‐1α, peroxisome proliferator‐activated receptor gamma coactivator‐1 alpha; ROS, reactive oxygen species; SCFAs, short‐chain fatty acids; SIRT1, sirtuin 1.

Pharmacological targeting of inflammatory pathways also presents therapeutic potential. Antiinflammatory and cytokine‐modulating agents may reduce neuroinflammation and its downstream effects on mitochondrial function and neuronal health. In addition, neuromodulator approaches such as vagus nerve stimulation and microbiota‐directed therapies are being explored for their ability to influence central nervous system function through the gut–brain axis [123, 124]. Although these approaches remain largely experimental, they represent promising avenues for future therapeutic development.

Lifestyle interventions, particularly regular physical exercise, provide additional nonpharmacological benefits. Exercise has been consistently associated with enhanced mitochondrial biogenesis, improved oxidative metabolism, reduced systemic inflammation, and increased gut microbial diversity, and the growth of beneficial bacteria (Figure 4). These combined effects contribute to improved cognitive outcomes for cancer survivors [121, 122].

**Figure 4:**
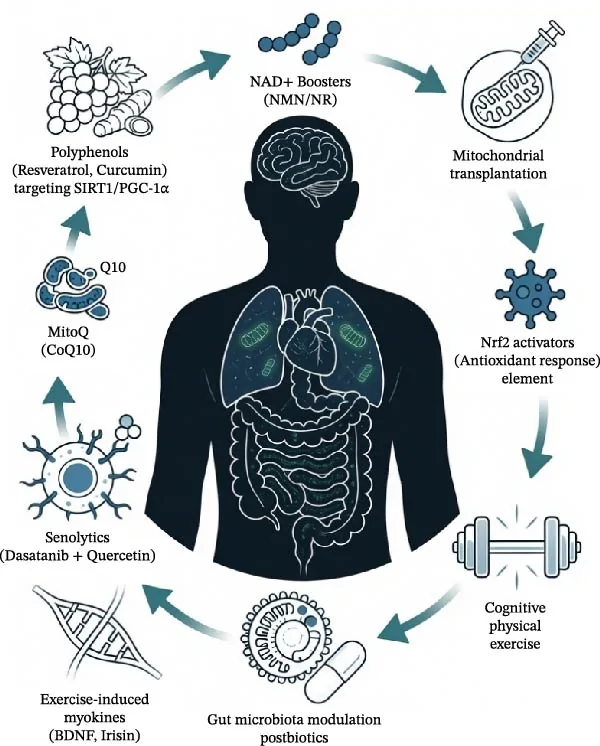
Multimodal therapeutic strategies for chemotherapy‐induced cognitive impairment. Interventions target mitochondrial function, the gut–brain axis, and neuroinflammation.

Overall, the complex pathophysiology of CICI necessitates a multimodal therapeutic approach integrating mitochondrial‐targeted strategies, microbiota modulation, dietary interventions, and lifestyle modifications, which may provide a more comprehensive approach to preserving cognitive function during and after chemotherapy. Future advances in personalized medicine will incorporate individual microbiome and metabolic profiles and mitochondrial biomarkers to optimize treatment outcomes [121, 125].

## 14. Current Limitations and Future Perspective

Although advances in understanding the mechanisms underlying CICI, several important challenges continue to limit the translation of experimental findings into clinical practice. Current evidence linking gut microbiota dysbiosis, mitochondrial dysfunction, and cognitive decline is derived predominantly from preclinical studies, with relatively limited validation in longitudinal clinical cohorts.

In addition, substantial heterogeneity exists across studies with respect to cancer types, chemotherapeutic regimens, treatment duration, experimental models, microbiome sequencing methodologies, and cognitive assessment tools, making direct comparisons difficult and limiting the identification of consistent biological signatures. Furthermore, increasing evidence supports an interaction between gut microbiota and mitochondrial homeostasis, but the temporal relationship and causal mechanisms underlying these processes remain incompletely understood.

Addressing these challenges will require well‐designed prospective clinical studies that integrate microbiome profiling, mitochondrial biomarkers, metabolomic analyses, neuroimaging, and standardized cognitive assessments. Such multidisciplinary approaches will be essential for establishing causal relationships, identifying reliable biomarkers, and determining whether mitochondrial reprogramming represents an early adaptive response or a maladaptive process driving the progression of CICI. A more comprehensive understanding of microbiota–mitochondria crosstalk may ultimately facilitate the development of personalized therapeutic strategies aimed at preserving cognitive function and improving the quality of life of cancer survivors.

## 15. Conclusion

CICI remains a significant challenge for cancer survivors, adversely affecting their quality of life. As highlighted throughout this review, accumulating evidence suggests that mitochondrial dysfunction and gut microbiota dysbiosis are closely interconnected through the gut–brain axis and collectively contribute to the pathogenesis of CICI. Within this framework, mitochondrial reprogramming emerges as a central adaptive process integrated by inflammatory, metabolic, and microbial signals, linking peripheral alterations with central nervous system dysfunction. Viewing CICI from this perspective highlights the complex interplay between systemic and central pathways while also identifying key targets for therapeutic intervention. Strategies aimed at restoring mitochondrial function and reestablishing microbial balance hold promise for improving cognitive outcomes.

Despite substantial progress in elucidating these mechanisms, much of the current evidence remains derived from preclinical studies. Future research should focus on translating these mechanistic insights into clinically relevant interventions. Well‐designed clinical trials incorporating personalized approaches based on individual microbiome and metabolic profiles are essential. Advances in understanding microbiota–mitochondria interactions are expected to drive the development of effective preventive and therapeutic strategies, ultimately improving the management of CICI in cancer survivors.

## Author Contributions

Conceptualization, supervision: Gayathri M. Rao. Literature search, original draft preparation: Gayathri M. Rao, Amisha Malhotra, Aradhana Marathe, and V. Samyuktha Rani. Data curation, figure preparation: Amisha Malhotra and Aradhana Marathe. Critical review, editing, final approval: Gayathri M. Rao, Sharada Rai, Ajeetkumar Patil, and Dinesh Upadhya, and M. Chakrapani.

## Funding

This research received no specific grant from any funding agency in the public, commercial, or not‐for‐profit sectors.

## Disclosure

All figures were critically reviewed, scientifically verified, and finalized by the authors.

## Conflicts of Interest

The authors declare no conflicts of interest.

## Data Availability

The data supporting the findings of this study are available from the corresponding author upon reasonable request.
